# Effectiveness of Link Prediction for Face-to-Face Behavioral Networks

**DOI:** 10.1371/journal.pone.0081727

**Published:** 2013-12-10

**Authors:** Sho Tsugawa, Hiroyuki Ohsaki

**Affiliations:** 1 Faculty of Engineering, Information and Systems, University of Tsukuba, Ibaraki, Japan; 2 School of Science and Technology, Kwansei Gakuin University, Hyogo, Japan; Semmelweis University, Hungary

## Abstract

Research on link prediction for social networks has been actively pursued. In link prediction for a given social network obtained from time-windowed observation, new link formation in the network is predicted from the topology of the obtained network. In contrast, recent advances in sensing technology have made it possible to obtain face-to-face behavioral networks, which are social networks representing face-to-face interactions among people. However, the effectiveness of link prediction techniques for face-to-face behavioral networks has not yet been explored in depth. To clarify this point, here we investigate the accuracy of conventional link prediction techniques for networks obtained from the history of face-to-face interactions among participants at an academic conference. Our findings were (1) that conventional link prediction techniques predict new link formation with a precision of 0.30–0.45 and a recall of 0.10–0.20, (2) that prolonged observation of social networks often degrades the prediction accuracy, (3) that the proposed *decaying weight* method leads to higher prediction accuracy than can be achieved by observing all records of communication and simply using them unmodified, and (4) that the prediction accuracy for face-to-face behavioral networks is relatively high compared to that for non-social networks, but not as high as for other types of social networks.

## Introduction

Research on link prediction for social networks has been actively pursued [1]–[8]. In link prediction for a given social network obtained from time-window observation, new link formation in the network is predicted from the topology of the observed network. A social network is represented as a graph where individuals are represented as nodes and social ties among them are represented as links. In the literature, several link prediction techniques have been proposed [4], [5], [9]–[11]. These techniques can be used to predict new link formation by estimating the likelihood of link formation between two nodes on the basis of the observed network topology. Social ties can be defined in a number of ways, and the accuracy of link prediction techniques has been investigated for several types of social networks such as coauthorship networks [3], email networks [12], and friendship networks [13]. Link prediction techniques are expected to be utilized for several applications such as recommendation [3], anomaly detection [14], network modeling [15], missing link detection [6], evaluation of network evolution mechanisms [16], reconstruction of networks [17], and classification of partially labeled networks [18], [19].

Recent advances in sensing technology have made it possible to obtain face-to-face behavioral networks, which are social networks representing face-to-face interactions among people [20]–[23]. For instance, in the SocioPatterns project, social networks representing face-to-face interactions among participants at an academic conference are constructed using badge-shaped sensing devices [20].

Link prediction for face-to-face behavioral networks should be useful for developing novel services and performing sociological analyses. Link prediction is promising for predicting communications that are likely to occur; this can be viewed as potential communication demands. Hence, link prediction in face-to-face behavioral networks may contribute toward realizing novel services such as friendship recommendation in real-world environments, which is already common in online environments. Link prediction techniques may also be useful for analyzing the evolutionary dynamics of social networks in real-world environments, an important topic in social science.

However, the effectiveness of link prediction techniques for face-to-face behavioral networks has not yet been fully explored. Our research group has been investigating the effectiveness of link prediction techniques for face-to-face behavioral networks using a publicly available dataset called the SocioPatterns dataset [20], which contains the history of face-to-face interactions among participants at an academic conference. Our preliminary results are presented in [24], [25]. In addition, Scholz *et al.* [27] experimentally investigated the accuracy of conventional link prediction techniques for face-to-face behavioral networks by using their own datasets. They also investigated the effectiveness of combining face-to-face behavioral networks with other types of social networks for link prediction [2]. To understand the effectiveness of link prediction techniques when applied to real-world social networks, however, more extensive investigation is necessary. For instance, the effects of the observation period of social networks on the prediction accuracy have not been discovered. The results in [1] should be verified using other datasets.

To clarify the effectiveness of link prediction techniques for face-to-face behavioral networks, here we investigate the accuracy of conventional link prediction techniques for networks obtained from the SocioPatterns dataset [20]. We extend our preliminary work [24] and investigate the prediction accuracy under various conditions. Our results support the findings in [1], and provide new findings, such as that incorporating temporal information about communication is essential for improving prediction accuracy. Moreover, we discuss the effectiveness of link prediction techniques for face-to-face behavioral networks compared with other networks through experiments with the Enron email dataset [26], which is one of the largest available email corpora.

The main contributions of this study can be summarized as follows.

- We investigated the prediction accuracy of conventional link prediction techniques for face-to-face behavioral networks by using publicly available datasets.
- We showed that in order to achieve high link prediction accuracy for face-to-face behavioral networks, it is essential to incorporate temporal information and to appropriately tune the length of the training period, the period spanned by the records analyzed; good tuning results in increased link prediction accuracy.
- We showed that the proposed *decaying weight* method can lead to higher prediction accuracy than can be achieved by observing all records of communication and simply using them unmodified. The prediction accuracy of the decaying weight method is comparable to the accuracy achievable with an appropriately tuned training period.

## Methods

### Link Prediction Techniques

Among various link prediction techniques proposed in the literature, the common neighbor (CN) [9], Adamic/Adar (AA) [10], preferential attachment (PA) [9], Jaccard coefficient (JC) [27], and resource allocation (RA) [11] are widely used and their accuracies have been explored for several types of networks [3], [12], [13], [28].

For each node pair 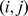, a link prediction technique gives 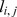, an estimate of the likelihood of link formation between nodes 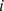 and 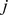. In other words, a link prediction technique predicts the likelihood of future link formation as 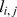.

In the following paragraphs, we briefly introduce definitions of the link prediction score 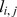 for the following types of link prediction techniques for unweighted and weighted networks: CN [9] and weighted CN (WCN) [4], [28], AA [10] and weighted AA (WAA) [4], [28], PA [9] and weighted PA (WPA) [28], JC [27] and weighted JC (WJC) [1], and RA [11] and weighted RA (WRA) [4]. In what follows, 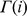 denotes the set of nodes adjacent to node 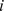, 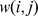 is a weight assigned to link 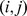, and 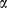 is a parameter for controlling the contribution of link weights to the link prediction score 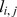.

#### Common neighbor

The common neighbor predicts new link formation from the idea that the existence of many common adjacent nodes between two nodes implies a high probability of new link formation between those two nodes [10]. In CN [10] and WCN [4], 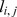, an estimate of the likelihood of link formation between node 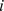 and 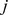, is given by
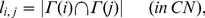
(1)

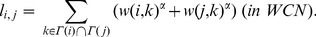
(2)


#### Adamic/Adar

The Adamic/Adar predicts new link formation from the idea that many common adjacent nodes with small degree between two nodes implies a high probability of new link formation between the nodes [10]. Similarly to CN, AA predicts new link formation on the basis of the number of common adjacent nodes, but assign a weight to 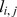 based on the degrees of common adjacent nodes. In AA [10] and WAA [4], 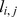 is given by
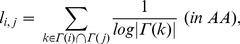
(3)

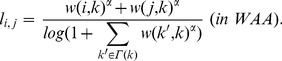
(4)


#### Preferential attachment

The preferential attachment predicts new link formation from the idea that a high-degree node has a higher chance of forming new links [9]. In PA [9] and WPA [4], [28], 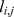 is given by
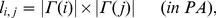
(5)

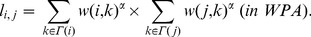
(6)


#### Jaccard coefficient

Similarly to CN, the Jaccard coefficient predicts new link formation from the number of common adjacent nodes, but the link prediction score is normalized [27]. In JC [27] and WJC [1], 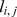 is given by
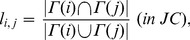
(7)

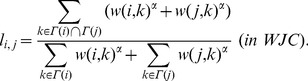
(8)


#### Resource allocation

The resource allocation predicts new link formation from an idea similar to that in AA [11]. In RA [11] and WRA [4], 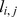 is given by
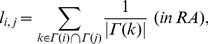
(9)

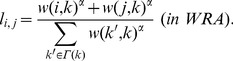
(10)


### Overview of Experiments

We evaluate the effectiveness of link prediction techniques through experiments using the SocioPatterns dataset [20], which contains the history of face-to-face communication among 110 participants over two and a half days at an academic conference (ACM Hypertext 2009). At that conference, face-to-face communication was detected and stored on radiofrequency identification (RFID) devices embedded in the conference badges worn by the participants. Badges periodically broadcasted ultra-low power radio packets that contained the participant’s ID. Each badge collected packets every 20 s, and stored a list of IDs and timestamps contained in the packets. Radio packet exchange was possible only when two people were close (1.0–1.5 [m] apart) and facing each other [20]. Face-to-face communication was detected by the IDs stored in the badges. An interval of 20 s is considered to be short enough to detect the occurrence of face-to-face communication at social gatherings [20].

For comparison purposes, we also use the Enron email dataset [36] that contains 252,759 email messages with headers and body texts exchanged between 151 employees of the Enron Corporation.

**Figure 1:**
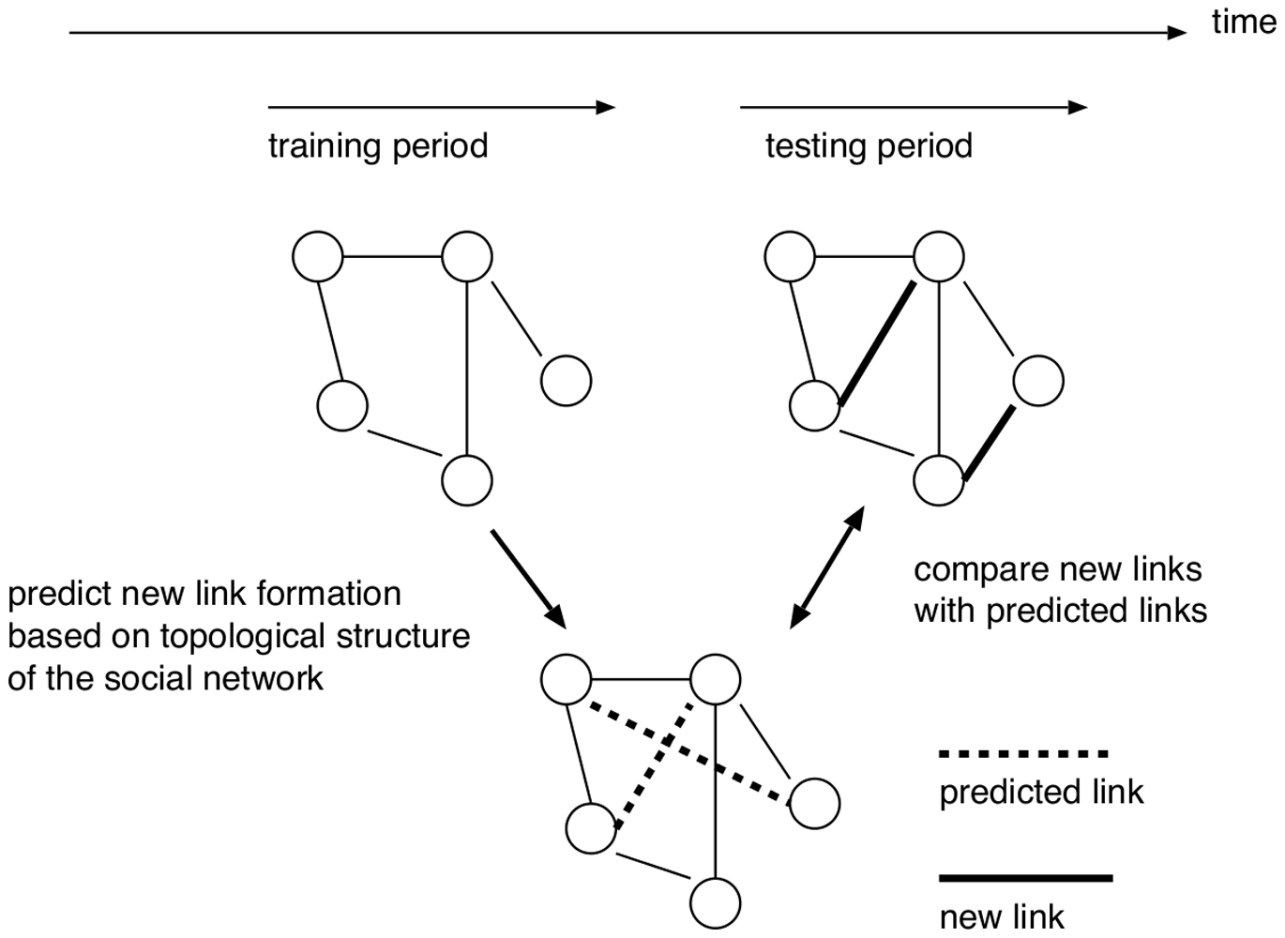
Overview of evaluation of link prediction techniques. New link formation is predicted from the topology of a social network obtained from communications in the training period. We investigate the accuracy of link prediction techniques by comparing the predicted links with actual new links created within the testing period.

In our experiments, we divide the history of communication into a *training period* and a *testing period*, and investigate how accurately the occurrence of communications in the testing period can be predicted using the history of communications in the training period. Figure 1 shows an overview of the evaluation method.

First, as an input for link prediction techniques, an undirected graph 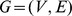, where each link weight is the number of communications, is obtained from the history of communication between times 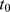 and 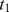. The period between 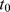 and 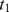 corresponds to the training period. In the graph 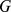, a link 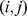 represents the existence of a communication between individuals 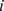 and 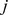 within the training period. The weight assigned to link 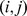 is defined as the number of communications between individuals 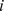 and 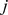 within the training period.

Next, we calculate the link prediction score 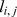 for each node pair 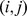 that is not associated with any communication before time 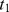 by using one of the link prediction techniques introduced in the previous section. Since 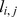 for CN, AA, PA, JC, and RA are defined for an unweighted graph, we obtain 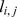 by simply ignoring link weights in 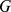.

As a final step, we examine how accurately we can predict which node pairs will have at least 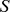 communications between times 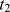 and 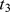 despite not having any communication before time 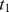. We perform this prediction by extracting node pairs whose link prediction scores 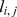 meet or exceed a threshold 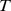. The period between times 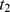 and 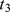 corresponds to the testing period. It is typical in studies of this kind to evaluate the accuracy of link prediction techniques for 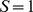 only [4], [28]. We introduce the parameter 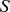, and evaluate the effectiveness of link prediction techniques not only for predicting new link formation but also for predicting the formation of strong ties (i.e., links with large weights).

The link prediction problem can be formulated as ranking or classification problems. Following prior work [1], [6], [13], [29]–[31], we use precision and recall, which are commonly used for evaluating ranking problems, as well as area under the receiver operating characteristic (ROC) curve (AUC) [32], which is commonly used for evaluation of classification problems. Precision 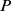 and recall 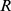 are defined as in Eqs. (11) and (12) using the concepts of true positive (TP), true negative (TN), false positive (FP), and false negative (FN). TP, TN, FP, and FN, which represent the respective numbers of node pairs satisfying the corresponding conditions in Table 1.
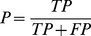
(11)

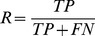
(12)


**Table 1:** Definitions of true positive (TP), true negative (TN), false positive (FP), and false negative (FN).

	Number of communications is morethan or equal to *S*	Number of communicationsis less than *S*
*l_i,j_* is larger than or equal to *T*	TP	FP
*l_i,j_* is smaller than *T*	FN	TN

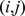
 satisfying the conditions shown in this table. TP, TN, FP, and FN are the numbers of node pairs

Precision evaluates the correctness, and recall evaluates the completeness of link prediction. Generally, there is a tradeoff between precision and recall, whereby a larger threshold 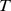 increases precision and decreases recall. AUC is defined as the area under the ROC curve [32]. The ROC curve is obtained by plotting the false positive rate (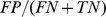) versus recall by changing the threshold 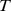. AUC is 0.5 when the prediction accuracy is equal to a random prediction, and is 1.0 with perfect prediction accuracy [32].

We perform experiments by changing 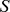 and 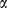. Unless explicitly stated we use 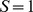 and 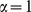. We first investigate the accuracy of conventional link prediction techniques for face-to-face behavioral networks using the SocioPatterns dataset, and then discuss the effectiveness of link prediction techniques for face-to-face behavioral networks compared to other networks through experiments with the Enron email dataset.

## Results and Discussion

### Characteristics of Datasets

**Figure 2:**
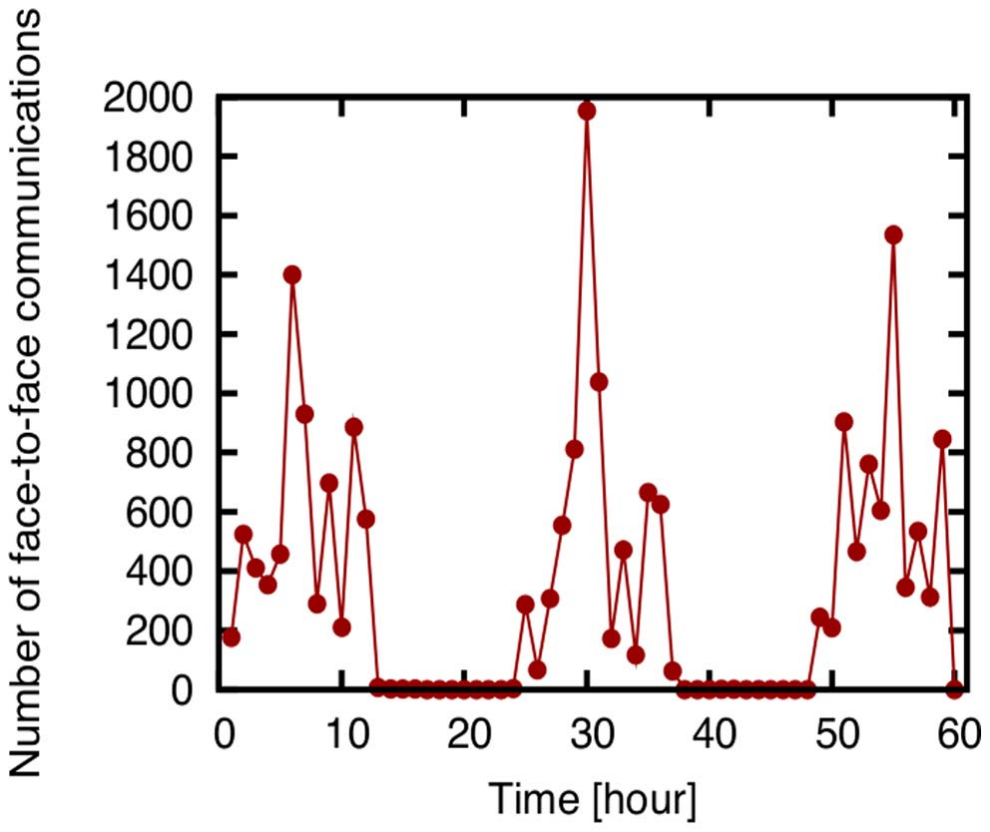
The number of face-to-face communications recorded in the SocioPatterns dataset over periods of one hour. The onset of sensing face-to-face interactions is indicated by 0.

**Figure 3:**
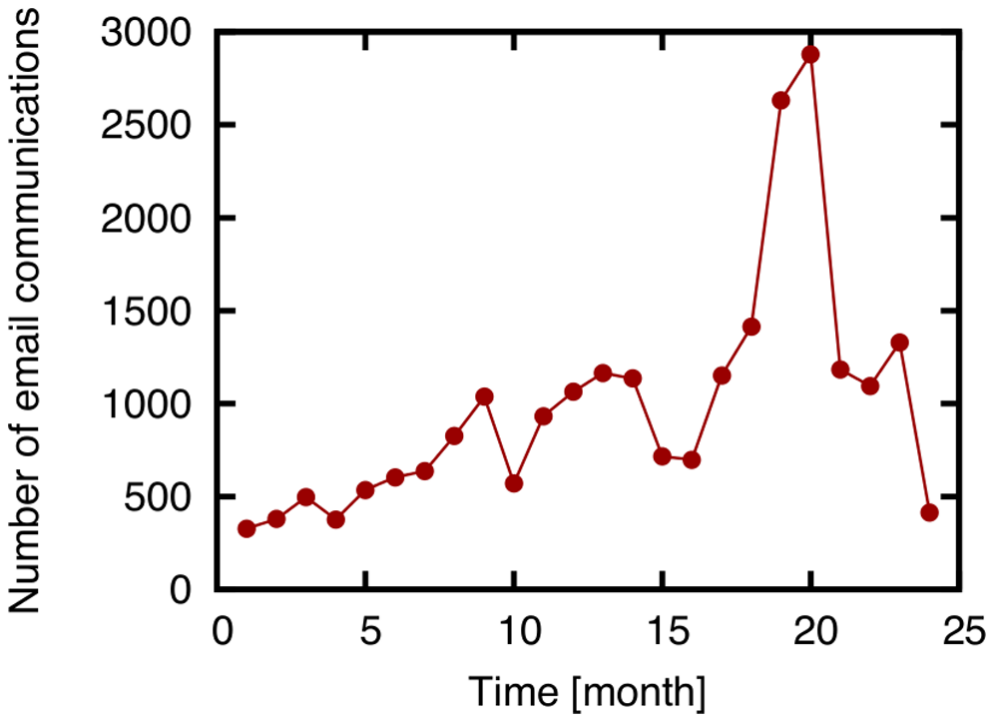
The number of email communications recorded in the Enron email dataset over periods of one month. The onset (indicated by 0) is April 1, 2000.

**Figure 4:**
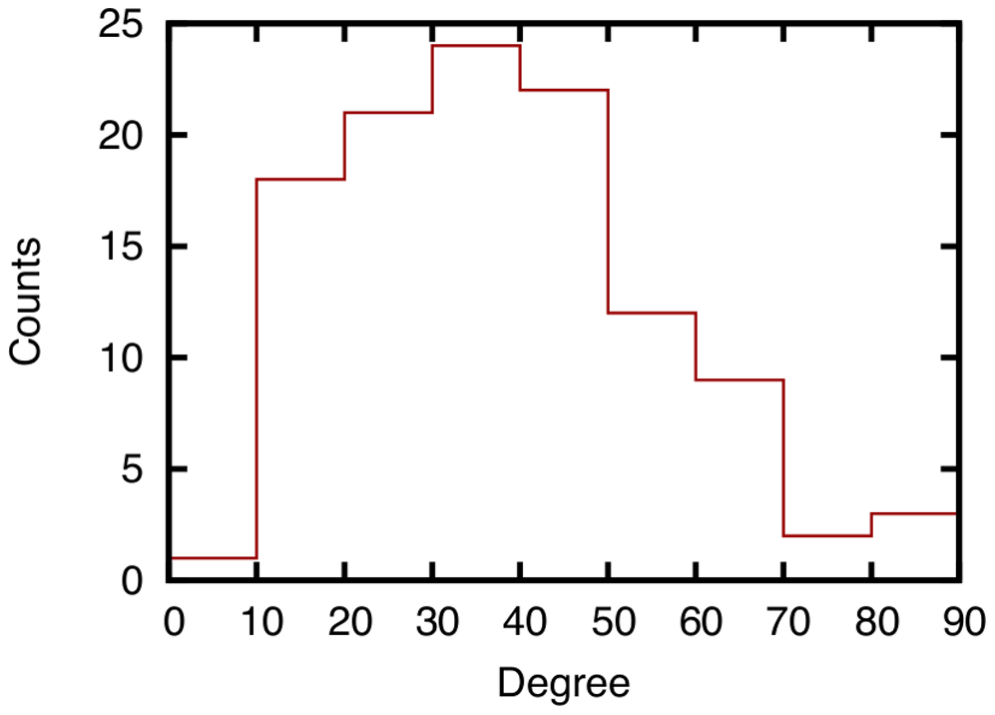
Degree distribution of a network obtained from the entire history of face-to-face communications in the SocioPatterns dataset. In the network, each node represents a participant at the conference, and each link between two nodes represents the occurrence of face-to-face communication between participants during the conference.

**Figure 5:**
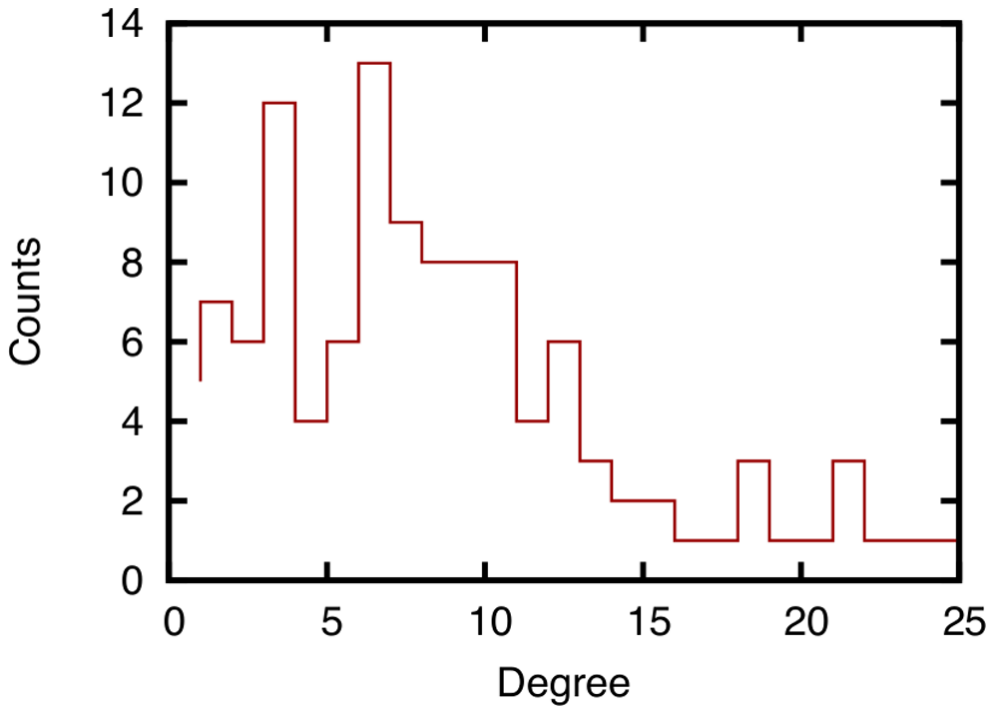
Degree distribution of a network obtained from the entire history of email communication in the Enron email dataset. In the network, each node represents an employee of the Enron Corporation, and each link between two nodes represents the occurrence of email communication between the corresponding employees from April 2000 to March 2002.

Before evaluating the effectiveness of link prediction techniques, we analyze the characteristics of datasets used in this paper. Figure 2 shows the number of face-to-face communications recorded in the SocioPatterns dataset over intervals of one hour, and Fig. 3 shows the number of email communications recorded in the Enron email dataset over intervals of one month. The degree distributions of networks constructed from all communication logs in the SocioPatterns dataset and the Enron email dataset are shown in Figs. 4 and 5, respectively.

Figure 2 shows that the number of face-to-face communications fluctuates in the SocioPatterns dataset, and Fig. 3 shows that there is no such tendency in email communications in the Enron email dataset. Figures 4 and 5 show that the degree distribution is vastly different in the two networks. For that reason, the effectiveness of link prediction techniques might be different for the two networks.

### Comparison of Ten Link Prediction Techniques

**Figure 6:**
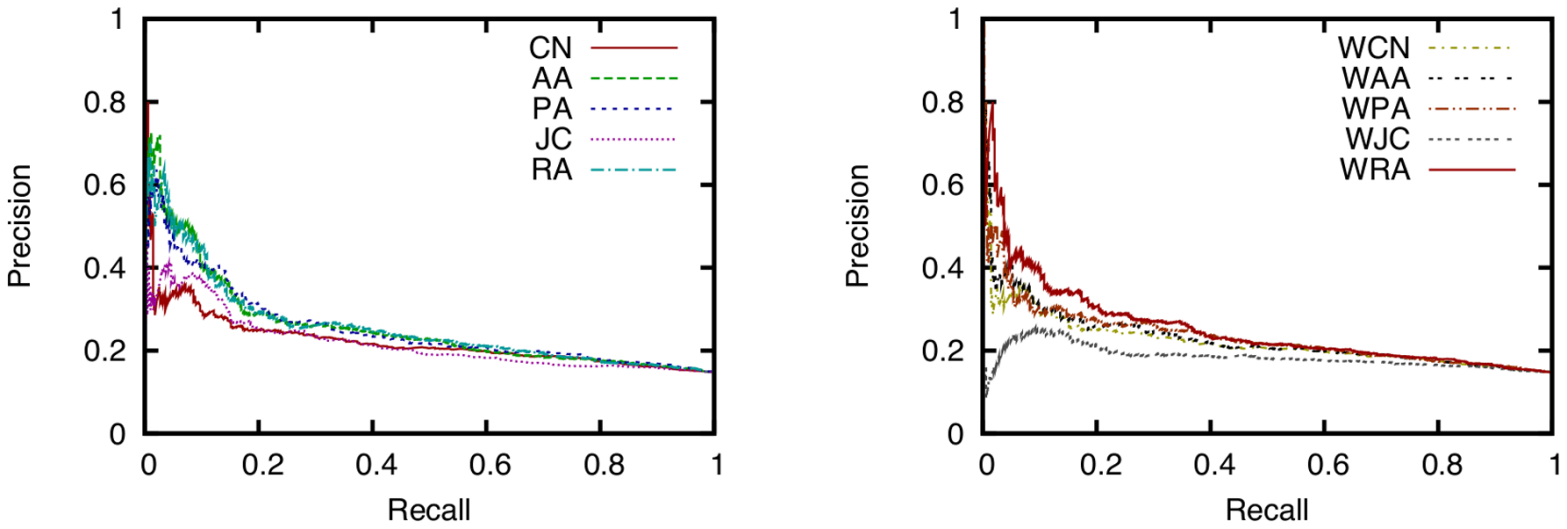
Relation between precision and recall for ten link prediction techniques. (training period: 0–30 (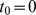, 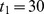), testing period: 30–60 (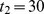, 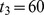), parameter for link prediction: 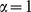, dataset: SocioPatterns dataset).

**Figure 7:**
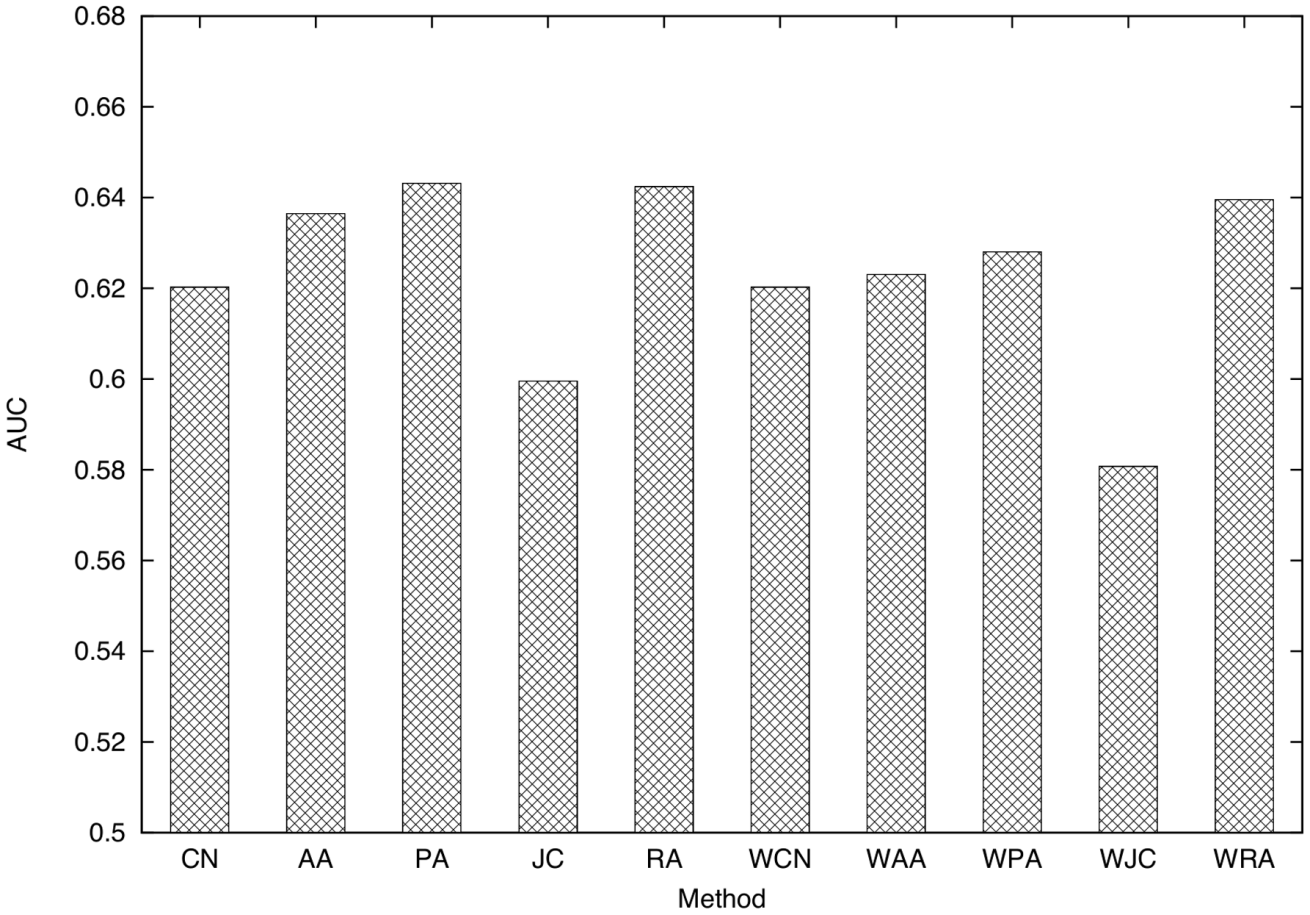
Comparison of the AUC scores of the ten link prediction techniques. (training period: 0–30 (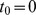, 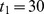), testing period: 30–60 (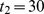, 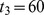), parameter for link prediction: 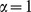, dataset: SocioPatterns dataset).

To investigate the accuracy of link prediction techniques for a face-to-face behavioral network, we find the precision and recall of ten link prediction techniques while varying the threshold 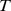. Precision-recall curves for ten link prediction techniques are shown in Fig. 6. The AUC scores of the ten link prediction techniques are shown in Fig. 7. The training period is time 0–30 [h] (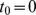, 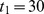) and the testing period is time 30–60 [h] (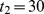, 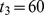).

Figure 6 shows that for instance, RA achieves a precision of 0.30–0.45 and a recall of 0.10–0.20 with an appropriate threshold 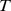 for a face-to-face behavioral network obtained from the SocioPatterns dataset.

Unsurprisingly, this result suggests that link prediction techniques cannot perfectly predict future link formation, but can nevertheless achieve considerable accuracy. It is therefore expected that conventional link prediction techniques can be applicable to services such as friendship recommendation that require a moderate level of prediction accuracy. For instance, it is reported in [29] that future friendship formation in an online social networking service called LiveJournal can be predicted with a precision of 0.18 and a recall of 0.18. Since networks in LiveJournal and the SocioPatterns dataset are rather different in terms of the number of nodes and links, direct comparison of the results for those two networks is impossible. However, we expect that a precision of 0.30–0.45 and a recall of 0.10–0.20 are sufficient accuracy for a friendship recommendation service.

Focusing on the differences among link prediction techniques, we find that RA and PA achieve the highest accuracy. Figure 7 shows that the AUC scores of RA and PA are the highest. The DeLong test [33] shows that the AUC scores of RA and PA are significantly higher than those for other methods (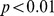), and that there is no significant difference between RA and PA (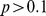). The high accuracy of RA is expected since the existence of many common neighbors between two individuals intuitively means a high probability of face-to-face communication. The higher accuracy of RA compared to CN and AA, which are similar to RA, is due to the existence of high-degree nodes (Fig. 4). RA successfully considers such high-degree nodes, which results in high prediction accuracy. In [11], it is shown that the performance of RA is higher than that of AA for networks containing nodes with notably high degrees. The high accuracy of PA is also due to the existence of high-degree nodes. Such nodes, which represent eminent participants at the conference, tend to have many communication events, which results in the high accuracy of PA. We can also see that the accuracies of techniques for the weighted networks (WCN, WAA, WPA, WJC, and WRA) are relatively lower than techniques for the unweighted networks (CN, AA, PA, JC, and RA). Lü *et al.* [4] report the same phenomenon, which is analyzed in greater depth in the next section by varying the parameter of the link prediction techniques.

### Effects of Link Prediction Parameters

**Figure 8:**
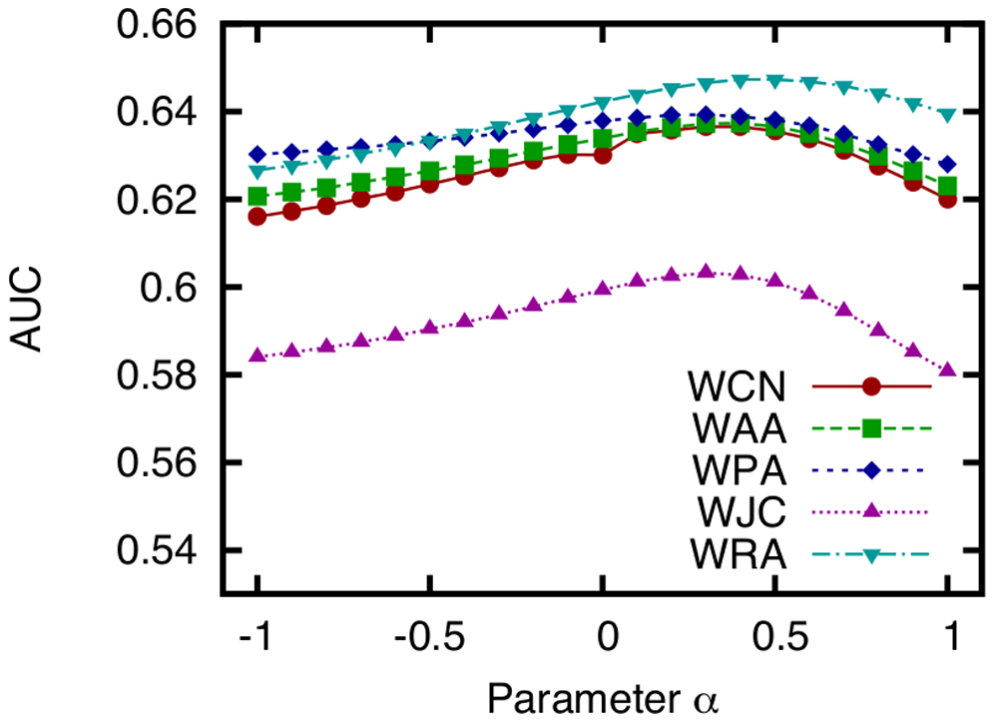
AUC scores of five link prediction techniques obtained while varying the parameter 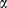, which controls the contribution of link weights to the link prediction score. (training period: 0–30 (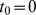, 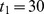), testing period: 30–60 (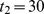, 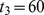), dataset: SocioPatterns dataset). 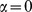 is equivalent to ignoring link weights, and 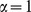 is equivalent to simply using the number of face-to-face communications as the link weight.

To investigate the effects of link prediction technique parameters on the prediction accuracy, we obtain the AUC scores of WCN, WAA, WPA, WJC, and WRA while varying the parameter 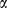, which controls the contribution of link weights to the link prediction score (Fig. 8).

This figure shows that appropriate link weights improve the accuracy of link prediction. However, as reported in [4], the prediction accuracy obtained when defining the link weight as the number of face-to-face communications and simply using it unmodified is even lower than that achieved when link weights are ignored altogether. All five techniques achieve the highest accuracy when 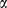 is approximately 0.3. This result suggests that the accuracy of link prediction can be improved by increasing the relative contribution of weak ties (links with small weights) to the link prediction score 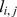. More detailed analysis is, however, required to determine the optimal value of 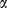.

### Effects of Social Tie Strength

**Figure 9:**
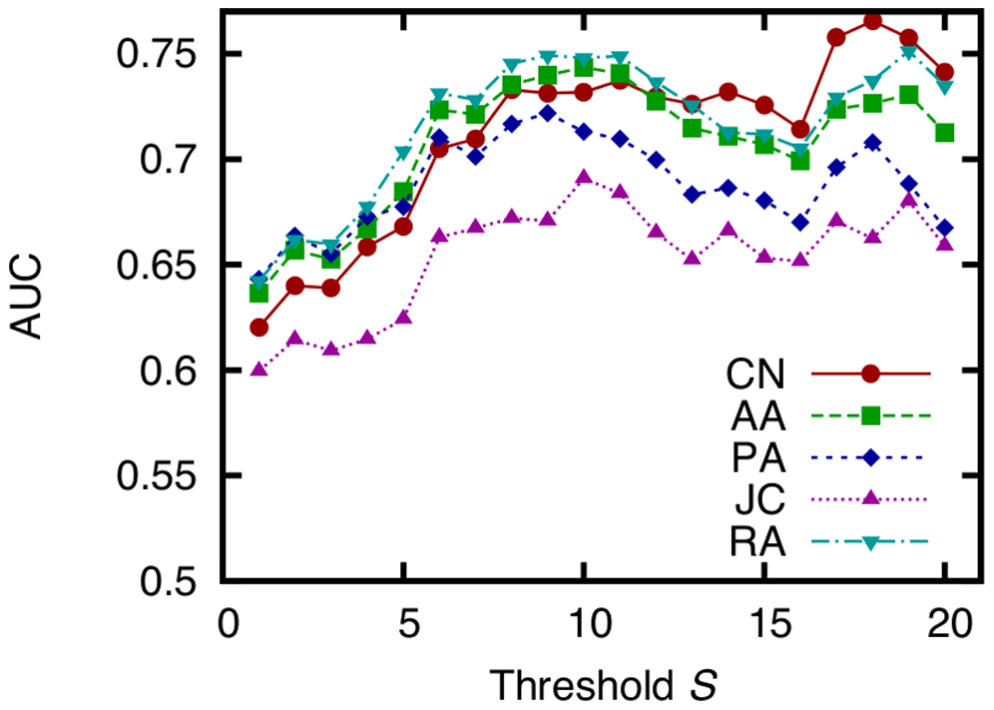
AUC scores of five link prediction techniques obtained while varying the threshold *S*. (training period: 0–30 (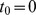, 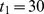), testing period: 30–60 (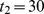, 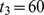), dataset: SocioPatterns dataset).

We next obtain the AUC score while varying the threshold 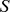 to investigate the accuracy of each technique to predict the formation of strong ties (links with large weights) (Fig. 9). In this investigation, the training period and the testing period are time 0–30 [h] and 30–60 [h], respectively. A large 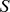 means that we predict only the formation of strong ties, and a small 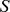 means that we predict the formation of links including weak ties (links with small weights). We show the results for only CN, AA, PA, JC, and RA in this investigation.

This figure shows that the accuracy is higher when predicting only the formation of strong ties than when predicting link formation including weak ties. This suggests that link prediction techniques are more effective at predicting the formation of strong ties than weak ties. This result is considered natural since weak ties may include noisy links, which are difficult to predict. Note that this tendency is observed in different face-to-face behavioral networks [1]. However, we should note that AUC scores do not change significantly when 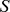 is six or higher.

### Effects of Training Period Length

In this section, we examine the effectiveness of using temporal information about communication in addition to the network topology for link prediction. By utilizing temporal information, we can change the contribution of each communication event to the link prediction score, which may result in more accurate prediction. A simple way to utilize temporal information is to change the training period length. By changing the onset time of the training period, we can evaluate the prediction accuracy when the contribution of old communications are ignored. The effect of the length of the training period on the prediction accuracy has not been fully explored in existing work [1] or our preliminary work [24], [25]. When applying link prediction techniques to face-to-face behavioral networks, it is important to know how to determine the training period.

**Figure 10:**
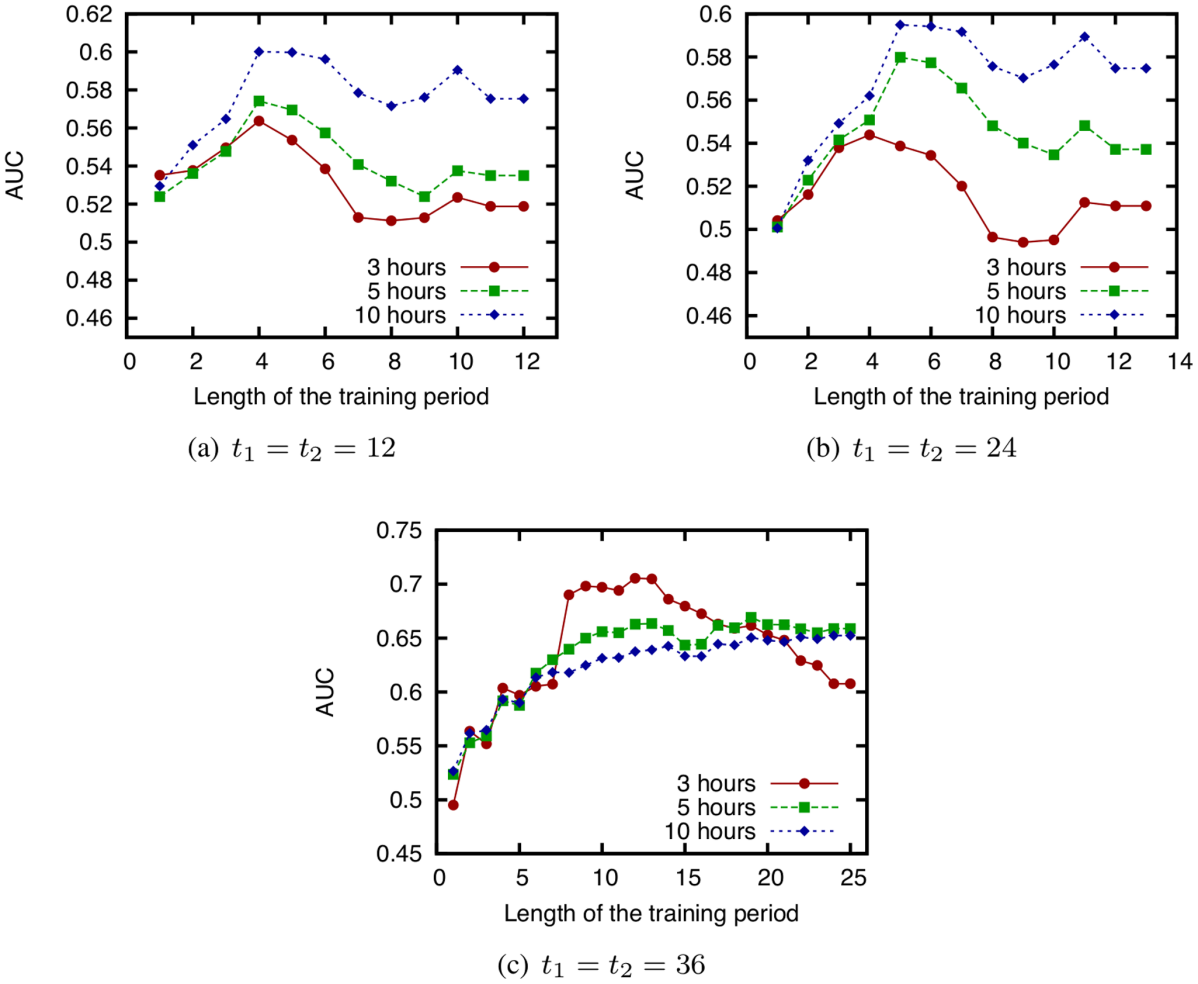
AUC score of CN obtained while varying the training period length. (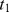 and 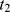 are fixed to 12, 24, or 36 [h], and 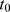 is varied. The testing period length is 3, 5, and 10 h. Dataset: SocioPatterns dataset).

We obtain the AUC scores of the link prediction techniques while varying the training period length for several starting times of the testing period 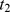 and lengths of the testing period. In this investigation, the testing period immediately follows the training period. Figure 10 shows the relation between the training period length and AUC score for CN. Note that the results when using other link prediction techniques are similar to the results shown in Fig. 10. Since few communications take place at times 12–24 and 36–48 [h] (see Fig. 2), we use the data for periods 0–12, 24–36, and 48–60 [h].

Figure 10 shows that simply increasing the training period length does not necessarily improve the prediction accuracy, and can even degrade it. Although it is intuitively expected that using more information should improve prediction accuracy, this result suggests otherwise.

Figure 10 also shows that the optimal training period length that maximizes the AUC score depends on the starting time of the testing period 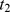. Note that the optimal training period length does not depend on the length of the testing period.

**Figure 11:**
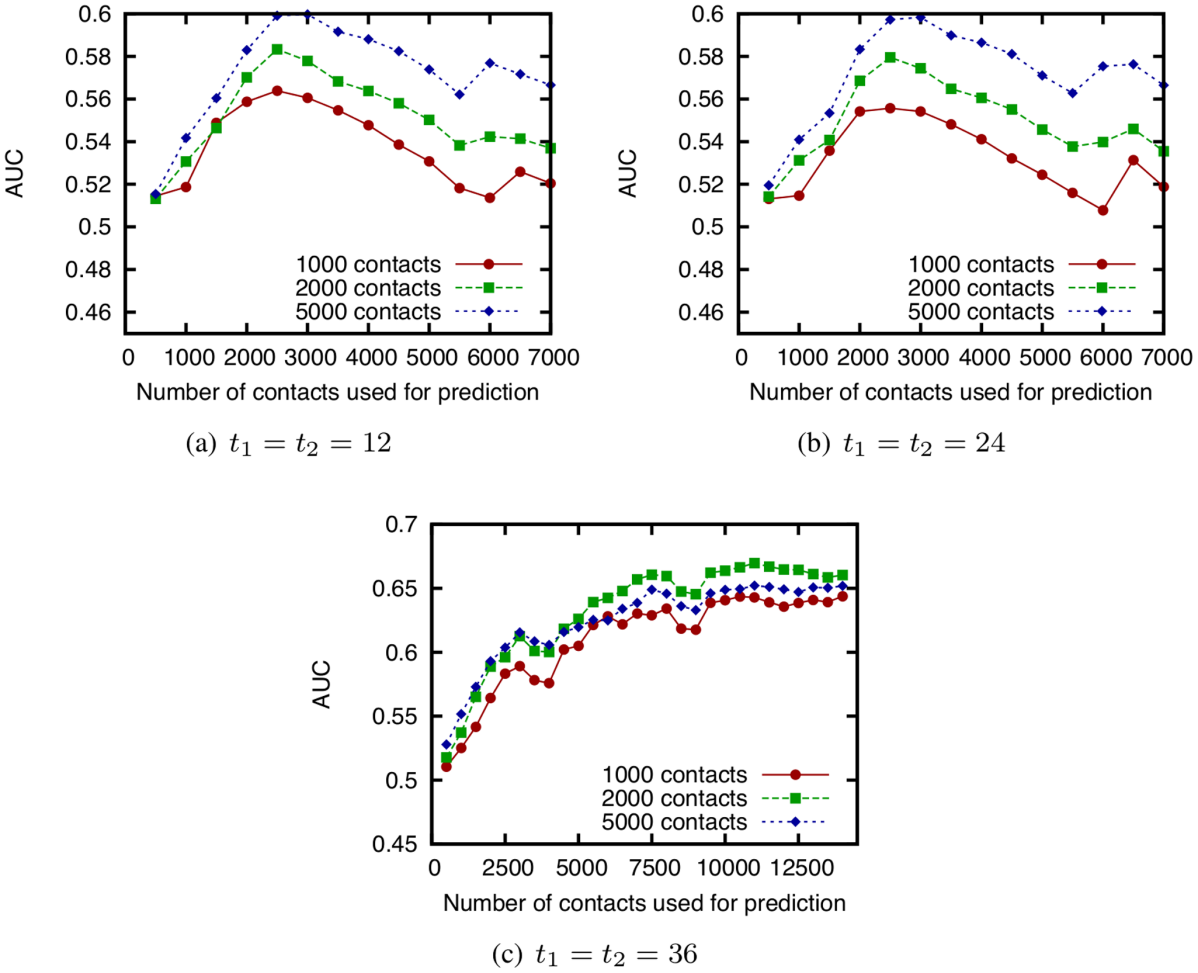
Relation between the number of face-to-face contacts used for link prediction and AUC score of CN. 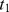
 and 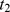 are fixed to 12, 24, or 36 [h], and 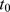 varies based on the number of contacts used for link prediction. 1000, 2000, and 5000 contacts are predicted immediately following the training period, using the SocioPatterns dataset.

We next determine the training period length not by time, but by the number of face-to-face contacts used for link prediction, and perform a similar experiment to investigate the effect of training period length. Since the number of face-to-face contacts recorded in the dataset is different from hour to hour, this may explain why the optimal training period length depends on the starting time of the testing period. Figure 11 shows the relation between the number of face-to-face contacts used for link prediction and AUC score. In this investigation, the testing period is also determined by the number of face-to-face contacts to predict.

These results show that in most cases, the prediction accuracy is highest when using approximately 3,000 contacts. However, when the starting time of the testing period is 36 [h], the prediction accuracy increases together with the number of contacts used for prediction is increased. We carefully investigated the results for other starting times in the testing period, and found that prediction accuracy increases as the number of contacts used for prediction increases when the testing period includes the third day. In other cases, using approximately 3,000 contacts achieves the highest accuracy.

These observations suggest that prolonged observation of face-to-face contacts for link prediction often degrades the prediction accuracy. We cannot draw general conclusions from our results alone, but in this experiment, using approximately 3,000 contacts (30 contacts per person) is optimal in many cases. Analysis using other datasets is required in order to determine the optimal training period length in the general case.

We next investigate the prediction accuracy when using a decaying weight method, which changes the contribution of each contact on the link prediction score by weighting each face-to-face contact. In the above experiments, all contacts used for link prediction are weighted equally for link prediction score calculation. However, our experimental results suggest that simply using all contacts often degrades the prediction accuracy. We therefore change the contribution of each contact on the link prediction score calculation by assigning a heavy weight to new information. In this experiment, we use two types of decaying weight methods, *linear* and *exponential*. Let contacts between time 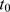 and time 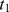 be 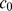, 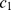, 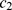,…, 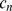. In the linear decaying weight method, for a contact 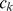 a link weight in the graph 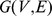, which is used for link prediction score calculation, is increased by 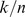, and in the exponential decaying weight method a link weight is increased by 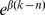. In exponential decaying weight method, 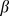 is determined as 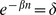, where 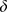 is a sufficiently small positive real number.

**Figure 12:**
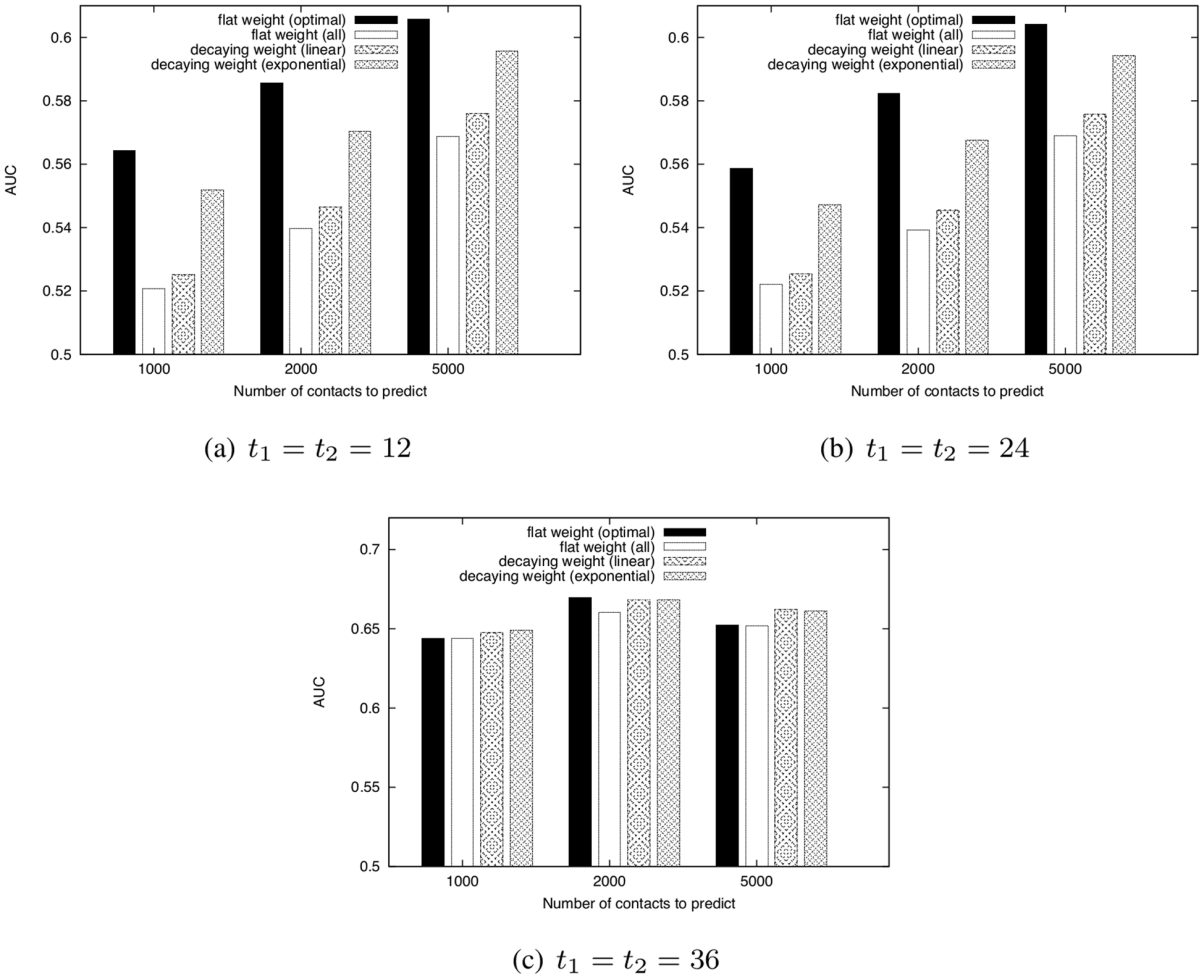
Comparison of the AUC scores of WCN for four types of weighting methods. 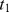
 and 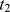 are fixed to 12, 24, or 36 [h]. 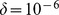. Parameter for link prediction: 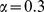, Dataset: SocioPatterns dataset.

Figure 12 shows AUC scores of WCN when using flat weight method, which equally weights all contacts, with the optimal length of the training period, flat weight method with 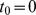, linear decaying weight method, and exponential decaying weight method.

These results show that the decaying weight method achieves higher accuracy than the flat weight method with 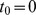. In particular, the accuracy of the exponential decaying weight method is comparable to that of the flat weight method with the optimal training period length. The DeLong test [6] shows that there are significant differences in AUC score between the exponential decaying weight method and the flat weight method with 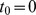 when 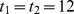 and 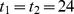 (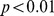). The DeLong test also shows that there are no significant differences in AUC score between the exponential decaying weight method and the flat weight method with the optimal training period length (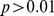). This result suggests that the decaying weight method can be useful when the optimal training period length is unknown.

The effectiveness of the decaying weight method is due to the spatiotemporal locality of face-to-face communication. The mobility of people is restricted by the space and their moving speed. Hence, if we know that person 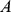 met person 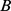, we can infer that 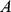 will tend to communicate with persons near 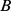 and not with persons located far away from 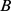. However, as time elapses, this information becomes irrelevant since persons 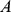 and 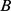 may move. Therefore, recent information is more important than old information, and the relevance of information about a particular time segment decreases with time.

### Comparison with the Enron Email Dataset

**Figure 13:**
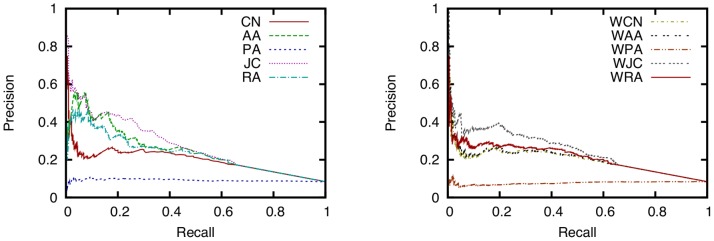
Relation between precision and recall of ten link prediction techniques. (training period: April 2000–March 2001, testing period: April 2001–March 2002, parameter for link prediction: 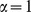, dataset: Enron email dataset).

**Figure 14:**
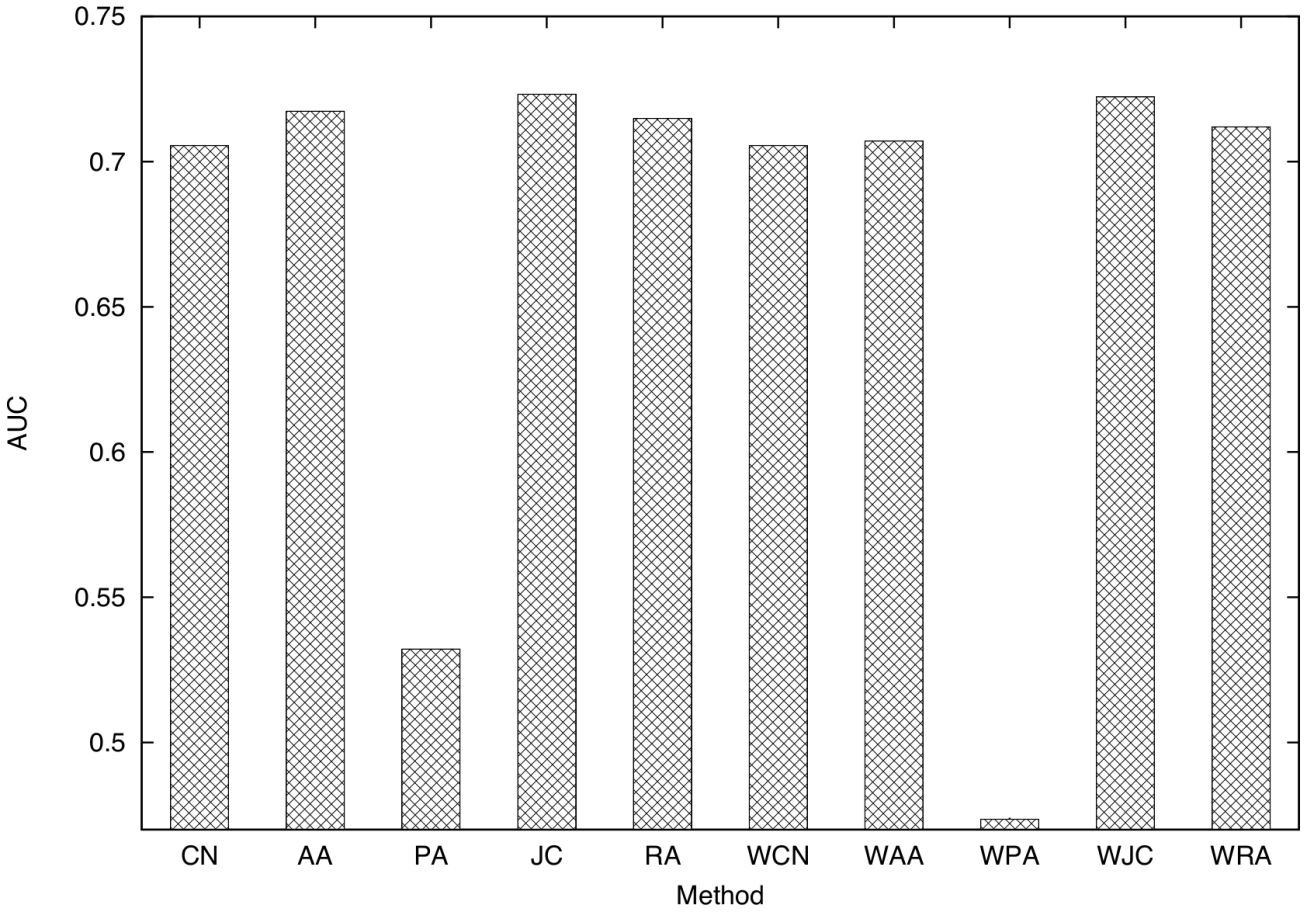
Comparison of the AUC scores of the ten link prediction techniques. (training period: April 2000–March 2001, testing period: April 2001–March 2002, parameter for link prediction: 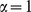, dataset: Enron email dataset).

We next compare the results for the SocioPatterns dataset and those for the Enron email dataset to investigate the characteristics of link prediction techniques for face-to-face behavioral networks. Figure 13 shows the relation between precision and recall for each link prediction technique for a network constructed from the Enron email dataset. Figure 14 shows the AUC scores of the ten link prediction techniques. In this investigation, the training period and the testing period are April 2000 to March 2001 and April 2001 to March 2002, respectively.

From Fig. 13, we find that, for instance, JC achieves a precision of 0.30–0.45 and a recall of 0.17–0.39 with an appropriate value of the threshold 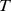 for an email network obtained from the Enron email dataset.

This result shows that conventional link prediction techniques achieve higher precision and recall for email networks than for face-to-face networks. Moreover, we should note that the number of nodes in the network obtained from the Enron email dataset is greater than that obtained from the SocioPatterns dataset. Hence, link prediction techniques have higher performance for email networks than for face-to-face networks. The topological structure of a network may affect the accuracy of link prediction techniques for the network.

Moreover, Fig. 14 shows that the accuracy of link prediction for the email network is significantly different among the ten link prediction techniques. The accuracy of PA and WPA for the network obtained from the Enron email dataset is significantly lower than that for other techniques. The DeLong test shows that there are significant differences in AUC score between PA and WPA and other techniques (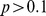). RA achieves the highest accuracy for networks obtained from the SocioPatterns dataset, but its accuracy is comparable to that of JC and AA for the network obtained from the Enron email dataset. The DeLong test shows that there are no significant differences in AUC score among AA, JC, and RA (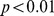). In the network obtained from the Enron email dataset, the number of high-degree nodes is smaller than that in the network obtained from the SocioPatterns dataset (Figs. 4 and 5). Therefore, the link prediction scores of RA tend to be similar to those of other similar techniques (i.e., AA and JC), which determines the similarity in accuracy between these techniques.

**Figure 15:**
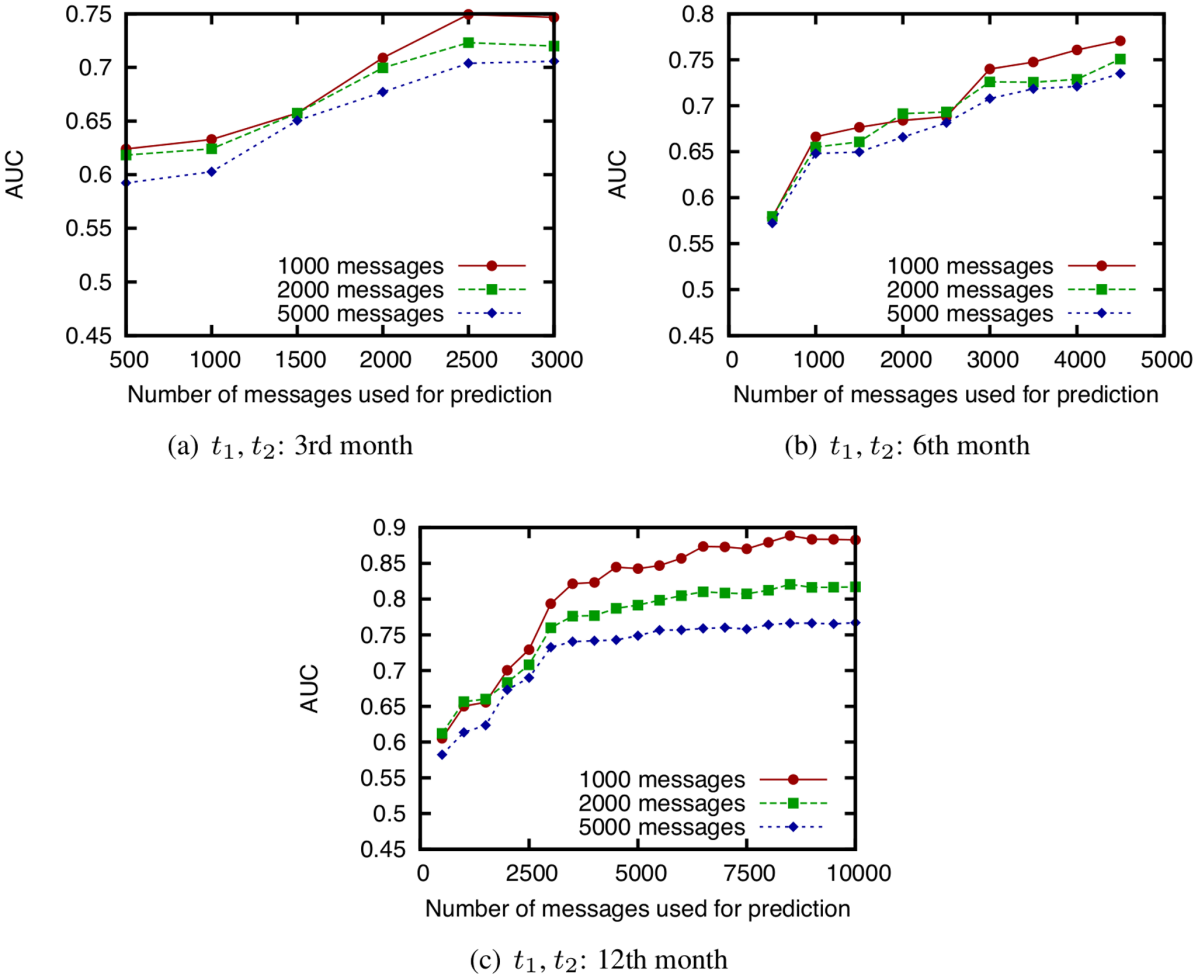
Relation between the number of email messages used for link prediction and the AUC score of CN. (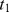 and 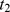 are fixed to the 3rd month, 6th month, or 12th month, and 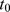 is changed based on the number of email messages used for link prediction. Predictions are made for 1000, 2000, and 5000 messages immediately after the training period. Dataset: Enron email dataset).

**Figure 16:**
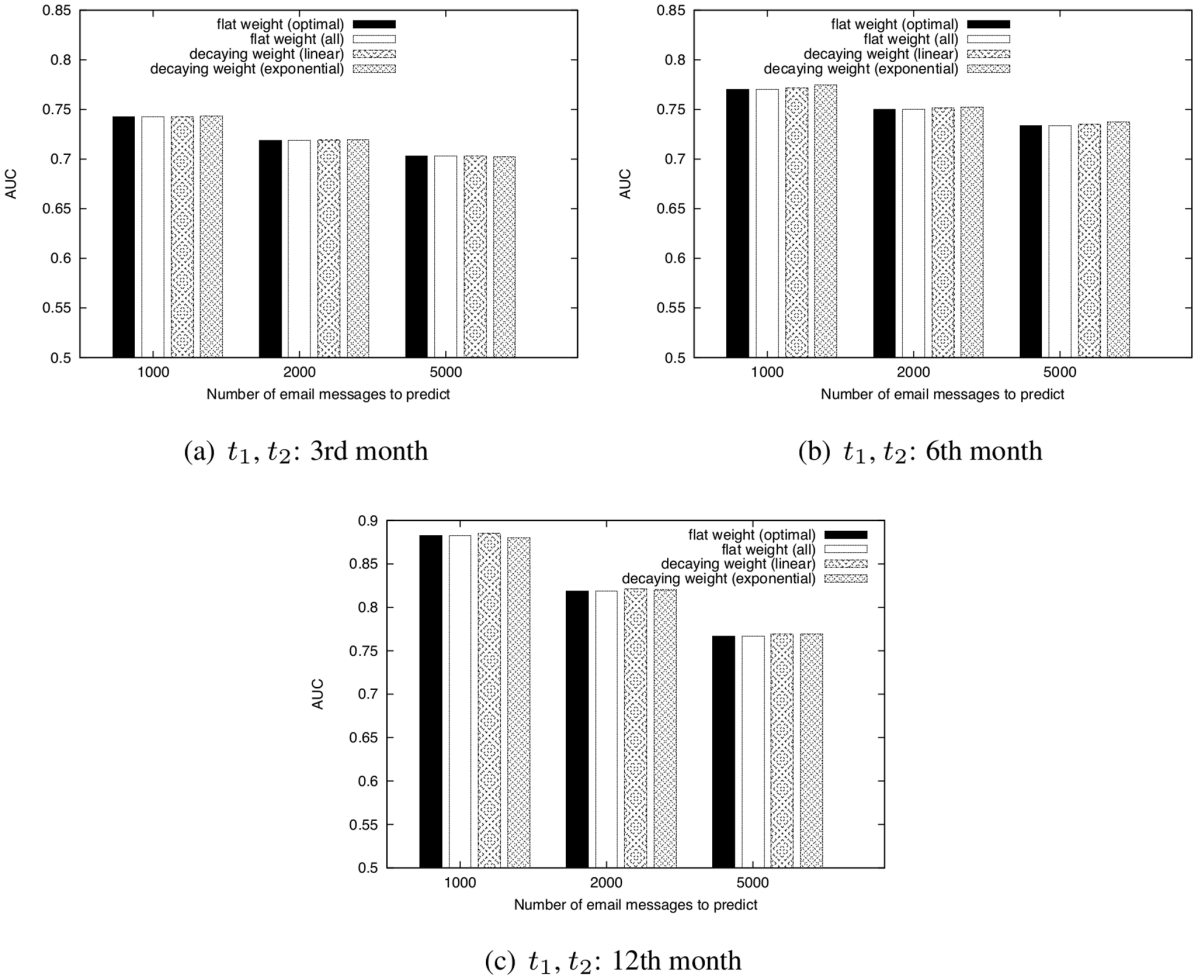
Comparison of the AUC scores of WCN for four types of weighting methods. 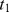
 and 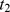 are fixed to the 3rd, 6th, or 12th month. 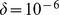. parameter for link prediction: 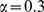, Dataset: Enron email dataset.

We next investigate the AUC score of CN for networks obtained from the Enron email dataset by changing the number of email messages used for prediction (Fig. 15). In this investigation, we compare the effectiveness of tuning the training period length for a face-to-face network and an email network. We also compare the AUC scores of each weighting method (Fig. 16) and investigate the effectiveness of incorporating temporal information with regard to link prediction.

Figure 15 shows that the prediction accuracy for an email network is improved when the training period length is extended. This tendency is different from the results for a face-to-face behavioral network (see Fig. 11), and suggests that the training period must be determined carefully in order to improve the prediction accuracy, particularly for a face-to-face behavioral network. Figure 16 shows that the decaying weight method is not effective, and that the naive method (i.e., the flat weight method) is sufficient for email networks. The DeLong test shows that there is no statistically significant difference between the AUC scores of the two methods (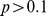). This result also suggests that, particularly for a face-to-face network, the training period should be tuned carefully to improve the prediction accuracy. As discussed in the previous subsection, in face-to-face networks, using temporal information about communications is important since face-to-face communication exhibits spatiotemporal locality. On the contrary, since email communication is not conducted in real time and email messages are exchanged over the Internet, the relevance of such locality for email networks is lower than that for face-to-face networks.

### Comparison with Other Networks

We lastly compare the prediction accuracy for a face-to-face behavioral network with that for other networks. Table 2 shows the structural characteristics of several types of networks and the AUC scores of CN for those networks. The AUC scores for terrorist, and protein, and food web networks are taken from Fig. 3 in [6], and that for Facebook user networks is taken from Tables 3 and 5 in [13].

**Table 2:** Structural characteristics of several types of networks and the AUC score of CN for those networks.

network	number of nodes	average degree	clustering coefficient	average path length	AUC
face-to-face (SocioPatterns dataset)	110	38.9	0.53	2.0	0.5–0.7
email (Enron email dataset)	151	9.3	0.70	6.0	0.6–0.85
terrorists [34]	–	4.9	0.36	2.6	0.5–0.8 [6]
Facebook [35]	63,731	24.3	–	–	0.8–0.94 [13]
protein [36]	–	4.8	0.06	3.7	0.5–0.58 [6]
food web [37]	–	3.0	0.17	3.3	0.5–0.7 [6]

Table 2 shows that CN achieves a higher AUC score for face-to-face networks than protein networks, but achieves similar or lower AUC scores compared to other networks. From this result, we conclude that the accuracy of link prediction techniques for face-to-face behavioral networks is higher than non-social networks, but not so high among other types of social networks. We plan to investigate the causes of this result in future research.

## Conclusions

To clarify the effectiveness of link prediction techniques for face-to-face behavioral networks, we have investigated the accuracy of conventional link prediction techniques for such networks under various conditions. We have performed several experiments utilizing the SocioPatterns dataset containing a history of face-to-face interactions among participants at an academic conference.

The experiments showed that conventional link prediction techniques predict new link formation in a face-to-face behavioral network with a precision of 0.30–0.45 and a recall of 0.10–0.20 through the use of an appropriate threshold. Comparison of the results for the SocioPatterns dataset and other datasets showed that the accuracy of link prediction techniques for face-to-face behavioral networks is relatively higher than that for non-social networks, but not particularly high among other types of social networks. These results suggest that conventional link prediction techniques are applicable to services, such as friendship recommendation, which require a moderate level of prediction accuracy.

The experiments also reveal a unique characteristic of link prediction for face-to-face behavioral networks whereby prolonged observation of social networks often degrades the prediction accuracy. For an email network, it is sufficient to use a naive method that observes all records of email messages and simply uses them unmodified. In contrast, for face-to-face behavioral networks, it is essential to determine an appropriate training period length to ensure high prediction accuracy. In most cases in our experiments, observing approximately 30 communications per person provided high prediction accuracy. However, further analysis is necessary in order to determine the optimal training period length in the general case.

One possible method that can be used to avoid the need for tuning the training period length in link prediction for face-to-face behavioral networks is using the decaying weight method. Our results showed that an exponential decaying weight method achieves comparable accuracy with the method using the optimal training period length, suggesting that the decaying weight method can be useful when the optimal length of the training period is unknown.

We plan to investigate the causes of differences in accuracy between different types of social networks. It is also important to investigate the optimal training period length in other face-to-face behavioral networks in future work.
